# Direct differentiation of cord blood derived mesenchymal stem cells into keratinocytes without feeder layers and cAMP inducers

**DOI:** 10.12669/pjms.36.5.1566

**Published:** 2020

**Authors:** Ayesha Kashmala Ghauri, Mohsin Wahid, Talat Mirza, Jahan Ara Ain Uddin

**Affiliations:** 1 Ayesha Kashmala Ghauri, Stem Cells and Regenerative Medicine Lab, Dow Research Institute of Biotechnology and Biomedical Sciences, Karachi, Pakistan; 2 Mohsin Wahid, Stem Cells and Regenerative Medicine Lab, Dow Research Institute of Biotechnology and Biomedical Sciences, Department of Pathology, Dow International Medical College, Dow University of Health Sciences (OJHA Campus), Karachi, Pakistan; 3 Talat Mirza, Department of Research, Ziauddin Medical University, Karachi, Pakistan; 4 Jahan Ara Ain Uddin, Department of Gynecology and Obstetrics, Dow University Hospital, Karachi, Pakistan

**Keywords:** Umbilical cord blood, Mesenchymal stem cells, Keratinocytes, Direct differentiation

## Abstract

**Objectives:**

The purpose of our study was isolation of umbilical cord blood derived mesenchymal stem (UCB-MSCs), their direct differentiation towards keratinocytes without using feeder layers, cAMP inducers and hormones known for morphological maintenance and proliferation of keratinocytes and characterization of UCB-MSCs through flowcytometry and keratinocytes through immunofluorescence.

**Methods:**

We have isolated and cultured UCB-MSCs (n=4) following critical parameters for successful isolation like sample processing within an hour of collection, gestational age not more than 38 weeks, no co-morbid and blood volume at least 80 ml. Cord blood mononuclear cells were isolated through ficoll based density-gradient centrifugation then cultured to isolate MSCs, defined by minimum criteria of International Society for Cellular Therapy. UCB-MSCs were then differentiated directly into keratinocytes. Differentiation was confirmed by morphology and characterized through immunofluorescence staining. UCB samples were collected from gynae/obstetric ward of OJHA campus under sterile conditions and processed at Stem cells and Regenerative medicine Lab, Dow Research Institute of Biotechnology and Biomedical Sciences, Ojha campus. The total duration of study was approximately 12 months.

**Results:**

We have successfully isolated UCB-MSCs that were plastic adherent, spindle shaped, showed trilineage mesodermal differentiation potential and were positive for CD90, CD73 and CD105 and negative for CD34 markers. UCB-MSCs were directly differentiated towards keratinocytes without using cAMP inducers, hormones or feeder layers. Differentiated keratinocytes attained typical honeycomb morphology and were stained positive on immunofluorescence for anti-pan cytokeratin antibody.

**Conclusion:**

Our study concludes possibility of direct differentiation of isolated and cultured UCB-MSCs into keratinocytes without using feeder layers and conventional keratinocyte culture media.

## INTRODUCTION

Mesenchymal stem cells (MSCs) are spindle shaped, adherent, multipotent tissue stem cells capable of self-renewal and differentiation into cells of multiple lineage. MSCs were first defined by Friedenstin et al. as fibroblast like cells which are able to differentiate into variety of cells like chondrocytes, osteocytes and adipocytes.^1^ The mesenchymal and tissue stem cell committee of the international society for cellular therapy proposed the minimum criteria that defines a cell as MSC. This include In-vitro plastic adherence of MSCs when maintained in standard culture conditions, tri-lineage differentiation potential of MSCs into adipocytes, chondrocytes and osteocytes and the cells must express positive markers CD73, CD90 and CD105, and negative markers CD45, CD34, CD14 or CD11b, CD79α or CD19 and HLA-DR surface molecules.^2^

MSCs can be isolated from various tissues like umbilical cord, adipose tissues, bone marrow, placenta, amniotic fluid, peripheral blood, endometrium, menstrual blood, breast milk, different dental sources and are present in almost all tissues. MSCs isolated from umbilical cord show primitive progenitor cells, high telomerase activity, high proliferation and differentiation capacity and minimal signs of aging^3^ which promises its usefulness in clinical therapies. MSCs from umbilical cord blood (UCB) are amongst the youngest with high proliferative potential cells but the yield of MSCs from UCB is very low when compared to bone marrow (gold standard).^4^ MSCs have different important roles in niches, wound healing, tissue repair, immunomodulation, in different diseases like hematological pathologies and malignancies, also in cardiovascular diseases, diabetes, renal diseases, hepatic injuries, autoimmune diseases and neurological disorders.^5^

Keratinocytes can be obtained from various sources, co-cultures, different feeder layers support or through differentiation of stem cells for clinical applications in regenerative medicine. Skin is the largest organ of the human body which acts as first line of defense. The main function of keratinocytes is to synthesize keratin which provides main mechanical support as they can grow and proliferate. Epidermis of the skin has the potential to constantly self-renew and proliferate. The maintenance of constant number of cells in a tissue is called tissue homeostasis is due to the presence of stem cells in the epidermis.^6^ Epidermal stem cells that are present in basal layer proliferate through basal cells of the inner most layer of epidermis and are connected to stratum basale through hemidesmosomes.

When culture in vitro morphology of human epidermal keratinocytes show honeycomb appearance, cohesive polygonal cells with granular cytoplasm.^7^ Some keratinocyte markers are keratin 19 (K19), K8, E-Cadherin, cytokeratin-7. K18 is primitive epithelial marker, p63 is epidermal precursor marker, K5, K14, K15 are basal keratinocyte markers, K1, K2, K10 (spinous layer), involucrin, filaggrin and loricrin (granular layer) are terminal differentiation and suprabasal keratinocyte markers.^8^

Different functions of epidermal keratinocytes include re-epithelialization after injury, barrier function and maintenance of epidermal homeostasis. Cultured keratinocytes can be used as cultured epithelial grafts, dermal substitutes, cultured keratinocytes can also be used in spray device systems for deep dermal burns treatment and also in aesthetical procedures and cosmetology.^9^,^10^

In this study we aimed at isolating Cord blood derived mesenchymal stem cells followed by their characterization using multicolor flowcytometery and tri-lineage differentiation of UCB-MSCs towards adipocytes, chondrocytes and osteocytes. The main objective of the study was to show the direct differentiation of MSCs towards keratinocytes without using feeder layers, hormones and cAMP inducers and characterization of keratinocytes using immunofluorescence staining for keratinocyte specific markers.

## METHODS

The consent form was designed in accordance to National Bioethics Committee Guidelines for Stem Cell Research in Pakistan. The study protocol and consent form was approved by Institutional Review Board of Dow University of Health Sciences (IRB No. IRB-739/767/DUHS/APPROVAL/2016/312). Four UCB samples were successfully processed for in-vitro study of isolation of MSCs from cord blood. UCB samples were collected from gynae/obstetric ward of OJHA campus and informed written consent was taken from mothers before sample collection. Samples were processed at Stem Cells and Regenerative medicine Lab, Dow Research Institute of Biotechnology and Biomedical Sciences, Ojha campus.

### Sample Collection

UCB was collected under sterile conditions, ex-utero after caesarean section. Placenta was collected immediately after delivery and blood was collected within 3–5 minutes of delivery of placenta in cord blood bags of 250 ml (811-2536, JMS). The sample processing was started within an hour of sample collection, in class II biosafety cabinet.

### Isolation of Cord Blood Mononuclear Cells (CBMNCs)

UCB was mixed with equal quantity of Dulbecco’s phosphate buffered saline (DPBS, 14190144, Thermofisher scientific), layered on top of ficoll (17-1440-03, GE healthcare) in ratio of 1:1:1. The tubes were centrifuged in swinging bucket centrifuge (Eppendorf, centrifuge 5810 R) at 800g for 30 minutes without brakes. Carefully aspirated the buffy coat and centrifuged at 800g for 10 mins. The supernatant was aspirated and the cells were washed with PBS. Once we got the pellet, cell count and viability analysis was done using cell viability analyzer (Vi-cell XR, Beckman Coulter).

### Primary Culture of MSCs

Approximately 1x10^8^ CB-MNCs with 98-100% total cell viability were plated in non-coated culture flasks/well plates in MSCs primary culture media as described in^11^ that comprise of Iscove’s modified Dulbecco’s medium (IMDM) (12440053), 10 ng/ml EGF (PHG0314), 10 ng/ml bFGF (PHG0024), 1% antibiotics-mycotics (15240062) (Thermofisher scientific) and 10-15% FBS (ES-009-C, embryo max speciality media). Cells were kept in 5% CO2 incubator at 37°C (New Brunswick Galaxy 170 R) for approximately 1hr and allowed to adhere. Cells were observed under microscope (Leica DMi1, Leica microsystems), non-adherent cells were removed and media in the flask was replaced. The media was changed after washing with 1X DPBS next day. Once the colony of adherent spindle shaped cells was appeared between day 7 to 15, media was changed every 4^th^ day with wash when needed. The cells were trypsinized with TrypLE express (12604013, Thermofisher scientific) or 0.25% trypsin-EDTA (T4049, Sigma Aldrich) when the colony reached 70 to 80% confluence. The cells were expanded and frozen till passage 4 (P4).

### Tri lineage mesodermal differentiation potential

P2 and P3 UCB-MSCs were used and cultured according to protocol of differentiation kits, osteocytes (A10072-01), chondrocytes (A10071-01), and adipocytes (A10070-01) all were bought from Gibco and were evaluated through Alizarin red stain, Alcacian blue stain and Oil red O stain solution respectively.

### Flowcytometry of UCB-MSCs

Immunophenotyping of UCB-MSCs was performed through flow cytometric analysis (BD FACS Celesta, software FACS Diva version 8) by using set of fluorochrome labelled antibodies against cell surface antigens. CD73, CD90 and CD105 as positive and CD 34 as negative markers (BD, Pharmingen). We have selected these antibodies from the set of negative and positive markers given in Dominici’s minimum criteria to define mesenchymal stem cells.^2^

### Direct differentiation of UCB-MSCs into keratinocytes

Approximately 20,000 P3 and P4 UCB-MSCs were plated in primary MSCs culture media. On reaching 70 to 80% confluence, media was replaced by pre warmed complete keratinocyte culture media that was prepared by primary culture media (PCM) and keratinocyte serum free media (K-SFM) as mentioned in.^12^ The protocol was modified as we prepared the media without using cAMP inducer like cholera toxin and hormones like insulin, tri-iodothyronine and hydrocortisone. PCM was prepared using DMEM Glutmax (10566016), HEPES 1M (15630106), 15% FBS. D-KSFM **(**10744019) and gentamicin (15710064) (Thermofisher scientific). The complete keratinocyte culture media was prepared by using PCM and K-SFM in the ratio of 1:9 and EGF 10 ng/ml and 2% antibiotic-mycotic solution. Media was changed every 2 to 3 days. UCB-MSCs started changing their morphology by 2^nd^ to 3rd day. Honeycomb appearance with polygonal to round shaped cells was observed between 3rd to 5^th^ day. 50 to 70% confluence of the cells was attained by day 8th to 11^th^. To characterize differentiated keratinocytes, immunofluorescence staining was performed.

### Immunofluorescence staining of differentiated keratinocytes

P3 UCB-MSCs were taken as negative control and anti-pan cytokeratin (AE1/AE3) alexafluor 488 antibody (53-9003-80, thermofisher scientific) was used to stain differentiated keratinocytes and DAPI for nucleus. Stained cells were visualized under fluorescent microscope Dmi8 using LAS v 4.8 (Leica Microsystem).

## RESULTS

### Primary culture of MSCs

**Fig.1:**
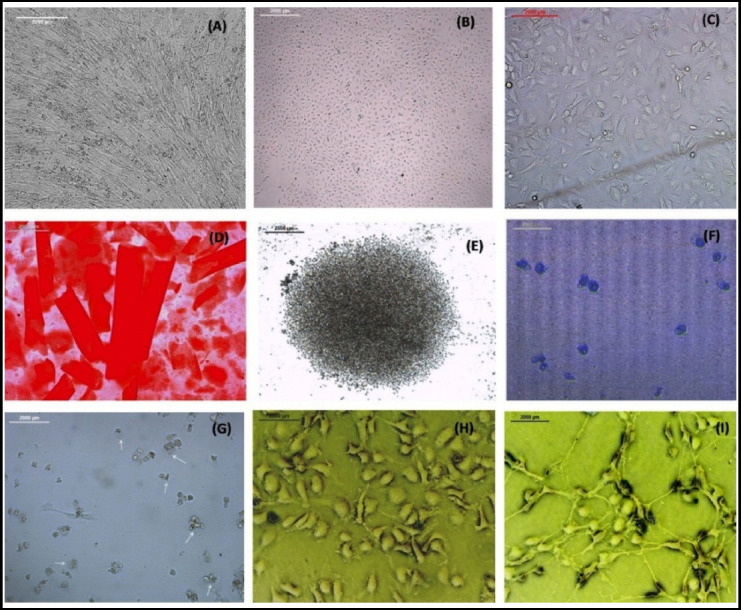
Spindle shaped UCB-MSCs magnification 10X. **(A)** Spindle to cuboidal shaped UCB-MSCs magnification 4X and 10X in **(B)** and **(C)** respectively. Osteogenic differentiation of UCB-MSCs at day 21, after alizarin red stain 10X. **(D)** Chondrogenic differentiation of UCB-MSCs at day 16 before stain 4X. **(E)** after alcacian blue stain 10X **(F)** Adipogenic differentiation of UCB-MSCs. **(G)** Direct differentiation of UCB-MSCs into keratinocytes day 3, polygonal cells with granular cytoplasm 20X **(H)** keratinocytes day 8, honey comb appearance of the cells 20X. **(I)** Scale bar of all parts of figure indicate 2000 um.

We got two different morphologies of UCB-MSCs from different cord blood samples (Fig.1 A-C). One is typical spindle shaped morphology. We got monolayers from these cells. After few passages the cells become enlarged and flattened with low proliferative potential. The other was comparatively smaller spindle to cuboid shaped. We found the latter cells to be more rapidly dividing. After few passages these cells grew in meshwork like pattern. The colony of MSCs appeared between 7 to 15 days, 70-80% confluence of the colony was attained in 22-27 days. On expansion UCB-MSCs showed high proliferative potential. We expanded UCB-MSCs till passage four for this study. 70-80% confluence on subsequent passages was attained in 2-16 days.

### Characterization of UCB-MSCs by flow cytometry

**Fig.2:**
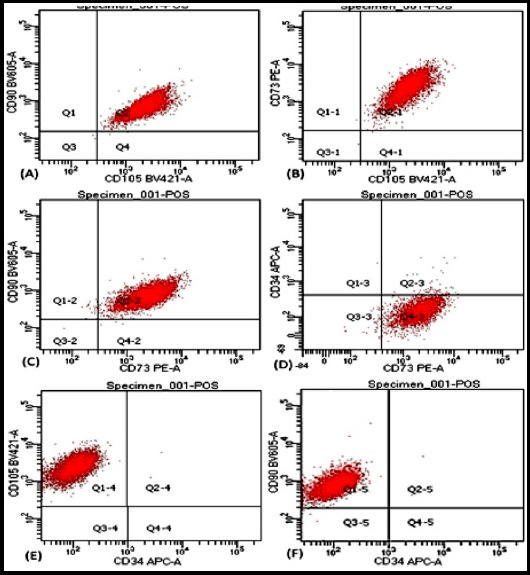
Flow cytometric analysis of UCB-MSCs stained sample. **(A)** the dot plot is double positive for CD 90 and CD 105, **(B)** for CD 73 and CD 105, and **(C)** CD 90 and CD 73. **(D)** The dot plot indicate UCB-MSCs are positive for CD 73 and negative for CD 34. **(E)** UCB-MSCs are positive for CD 105 and negative for CD 34. **(F)** UCB-MSCs are positive for CD 90 and negative for CD 34.

The results showed that UCB-MSCs were positive for CD73, CD90 and CD105 and negative for CD34 antibodies (Fig.2).

### Trilineage mesodermal differentiation

P2 and P3 UCB-MSCs were used for this part of study. Osteogenic differentiation was attained in approximately 21 days and was confirmed by positive alizarin red stain (Fig.1D).

UCB-MSCs differentiation into chondrocytes was confirmed at day 16^th^ with positive alcacian blue staining indicated the presence of proteoglycans, cartilage specific protein (Fig.1 E-F). For adipogenic differentiation, MSCS changed their morphology to more circular cells (Fig.1G). Cells were stained with Oil red O stain on day 8^th^ but no lipid filled cells were seen stained on microscopy. As reported previously, mostly UCB-MSCs show no to impaired adipogenic differentiation potential.^4^

### Differentiation of UCB-MSCs into keratinocytes

P3 and P4 UCB-MSCs were plated in MSCs primary culture media till cells attained 70-80% confluence. When complete keratinocyte media comprising of PCM and defined K-SFM was replaced, UCB-MSCs started changing their morphology next day, by day 5-8 honeycomb appearance, cohesive polygonal cells with granular cytoplasm were observed, 50–70% confluence of the cells reached in 8 to 11 days (Fig.1 H-I).

### Characterization of keratinocytes through immunofluorescence

**Fig.3:**
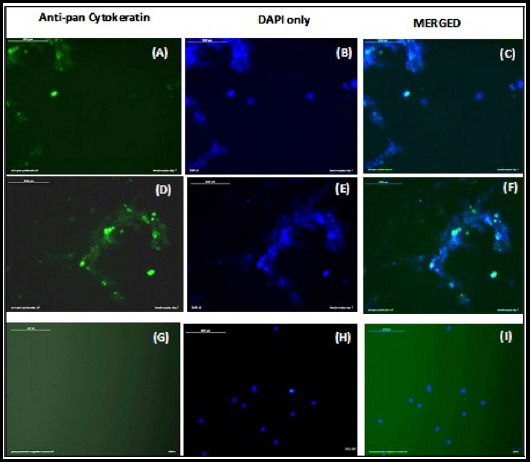
**(A, D)** differentiated keratinocytes are positive for green color stain of anti-pan cytokeratin. **(G)** P2 UCB-MSCs were taken as control were negative for anti-pan cytokeratin, no green color stain. **(B and E)** Differentiated keratinocytes showed blue color positive for DAPI. **(H)** P2 UCB-MSCs were positive for DAPI indicated with blue color. **(C and F)** merged images of anti-pan cytokeratin and DAPI for keratinocytes. **(I)** merged image of anti-pan cytokeratin and DAPI for UCB-MSCs control. All the above images are confirming that the UCB-MSCs are successfully differentiated towards keratinocytes. Magnification 20X.

Keratinocytes showed green color stain of the anti-pan cytokeratin and blue color for DAPI, whereas MSCs as control were negative for anti-pan cytokeratin and positive for DAPI confirmed the successful direct differentiation of UCB-MSCs towards keratinocytes. Cells were observed under inverted phase contrast fluorescent microscope Leica Dmi8 using LAS v 4.8 (Leica Microsystem) (Fig.3).

## DISCUSSION

This study will help the researchers to show the possibility of direct differentiation which will be simple, cost effective technique and a stepping stone to link between in vitro and in vivo studies that shall be helpful in future clinical trials related to pathological diseases aiming wound repair and tissue regeneration and cosmetology. As per our literature review there is no study in Pakistan about isolation of MSCs from cord blood and its direct differentiation towards keratinocytes although few studies have shown isolation of MSCs from different sources like mice bone marrow or human placenta.^13^-^15^ As previously published,^4^ we followed the important parameters for isolation and culture of UCB-MSCs from CBMNCs. On microscopy we have got two different morphologies of MSCs, typical spindle shaped and comparatively small rapidly dividing spindle to cuboid shaped cells (Fig.1 A-C). We confirmed the isolation of UCB-MSCs through Dominici’s criteria^2^ spindle shaped morphology, high proliferative potential and plastic adherent nature of cells, characterization through flow cytometry showed positive CD73, CD90 and CD105 and negative hematopoietic marker CD34 (Fig.2) and mesodermal differentiation potential into osteocytes, chondrocytes and adipocytes (Fig.1 D-G).

A published study showed the possibility of differentiation of UC-MSCs into dermal fibroblast like cells and AECS (amniotic epithelial cells) into skin like cells.^15^ Previously in a study Toai’s et al. showed differentiation of hUCB-MSCs into keratinocytes using cholera toxin and hydrocortisone as components of complete media of cell culture.^12^ In the last decade serum free, feeder free techniques have been developed to culture Keratinocytes.^16^ Role of cAMP inducers like cholera toxin or isoproterenol that effects proliferative potential of keratinocyte cultures in vitro is critical.^17^

In contrast to other studies we directly differentiated UCB-MSCs towards keratinocytes by using specific combination of media without feeder layers, cAMP inducers like cholera toxin and isoproterenol or hormones like hydrocortisone, insulin or triiodothyronine. Keratinocytes attained morphology of adhesive polygonal shaped cells and honeycomb appearance by day 3-5 (Fig.1 H-I) of direct differentiation. On 8^th^ day we confirmed successful differentiation of UCB-MSCs towards keratinocytes by positive immunofluorescence staining using anti pan cytokeratin (AE1/AE3) antibody which is cocktail of pan anti-cytokeratins (Fig.3).

### Limitations of the study

Immunofluorescence staining for characterization of differentiated keratinocytes could be performed on more samples but due to time and budget constraints it was not possible.

## CONCLUSION

UCB-MSCs have potential to directly differentiate into keratinocytes without using feeder layers, cAMP inducers and hormones by culturing in specified culture media.

### Author’s Contributions

**MW:** Designed and supervised the M. Phil Student Project. All lab work including Cell Culture, Flow cytometry and Immunofluorescence was done under his supervision. He is responsible and accountable for the accuracy or integrity of the work.

**AKG:** M. Phil student who carried out the lab work under supervision.

**TM:** Co supervisor for the project.

**JAH:** Facilitated the cord blood collection.

## References

[1] Friedenstein A, Deriglasova U, Kulagina N, Panasuk A, Rudakowa S, Luria E (1973). Precursors for fibroblasts in different populations of hematopoietic cells as detected by the in vitro colony assay method. Exp Hematol.

[2] Dominici M, Le Blanc K, Mueller I, Slaper-Cortenbach I, Marini F, Krause D (2006). Minimal criteria for defining multipotent mesenchymal stromal cells. The International Society for Cellular Therapy position statement. Cytotherapy.

[3] Vidal MA, Walker NJ, Napoli E, Borjesson DL (2011). Evaluation of senescence in mesenchymal stem cells isolated from equine bone marrow, adipose tissue, and umbilical cord tissue. Stem Cells Dev.

[4] Bieback K, Netsch P (2016). Isolation, Culture, and Characterization of Human Umbilical Cord Blood-Derived Mesenchymal Stromal Cells. Mesenchymal Stem Cells:Methods Mol Biol.

[5] Miura Y (2016). Human bone marrow mesenchymal stromal/stem cells:current clinical applications and potential for hematology. Int J Hematol.

[6] Liu N, Matsumura H, Kato T, Ichinose S, Takada A, Namiki T (2019). Stem cell competition orchestrates skin homeostasis and ageing. Nature.

[7] Mahabal S, Konala VBR, Mamidi MK, Kanafi MM, Mishra S, Shankar K Sequential cultivation of human epidermal keratinocytes and dermal mesenchymal like stromal cells in vitro. Cytotechnology.

[8] Du H, Wang Y, Haensel D, Lee B, Dai X, Nie Q (2018). Multiscale modeling of layer formation in epidermis. PLoS Comput Biol.

[9] Hirsch T, Rothoeft T, Teig N, Bauer JW, Pellegrini G, De Rosa L (2017). Regeneration of the entire human epidermis using transgenic stem cells. Nature.

[10] Johnstone P, Kwei JS-S, Filobbos G, Lewis D, Jeffery S (2017). Successful application of keratinocyte suspension using autologous fibrin spray. Burns.

[11] Van Pham P, Vu NB, Pham VM, Truong NH, Pham TL-B, Dang LT-T (2014). Good manufacturing practice-compliant isolation and culture of human umbilical cord blood-derived mesenchymal stem cells. J Transl Med.

[12] Toai TC, Thao HD, Gargiulo C, Thao NP, Thuy TTT, Tuan HM (2011). In vitro culture of Keratinocytes from human umbilical cord blood mesenchymal stem cells:The Saigonese culture. Cell Tissue Bank.

[13] Shaer A, Azarpira N, Aghdaie MH, Esfandiari E (2014). Isolation and characterization of Human Mesenchymal Stromal Cells Derived from Placental Decidua Basalis;Umbilical cord Wharton's Jelly and Amniotic Membrane. Pak J Med Sci.

[14] Mortazavi SM, Shekoohi-Shooli F, Aghamir SM, Mehrabani D, Dehghanian A, Zare S (2016). The healing effect of bone marrow-derived stem cells in acute radiation syndrome. Pak J Med Sci.

[15] Mahmood R, Choudhery MS, Mehmood A, Khan SN, Riazuddin S (2015). In Vitro Differentiation Potential of Human Placenta Derived Cells into Skin Cells. Stem Cells Int.

[16] Tjin MS, Chua AWC, Tryggvason K (2020). Chemically defined and xenogeneic-free culture method for human epidermal keratinocytes on laminin-based matrices. Nat Protoc.

[17] Takagi R, Yamato M, Murakami D, Kondo M, Yang J, Ohki T (2011). Preparation of keratinocyte culture medium for the clinical applications of regenerative medicine. J Tissue Eng Regen Med.

